# Case report of Zika virus during controlled ovarian hyperstimulation: results from follicular fluid, cumulus cells and oocytes

**DOI:** 10.5935/1518-0557.20180081

**Published:** 2019

**Authors:** Edilberto Araújo Filho, Cássio Leão Fácio, Ligiane Alves Machado-Paula, Mariana Angelozzi de Oliveira, Ciro Dresch Martinhago, Leonardo Previato Araújo, Lígia Fernanda P. Araújo

**Affiliations:** 1 Center of Human Reproduction of São José do Rio Preto, São José do Rio Preto – SP, Brazil; 2 Chromosome Genomic Medicine, São Paulo – SP, Brazil

**Keywords:** Zika virus, *in vitro* fertilisation, follicular fluid, oocytes, RT-PCR

## Abstract

We describe a case of a 37-year-old female, indicated for *in vitro* fertilisation. She developed skin rash on her trunk and limbs, during the treatment. RT-PCR results were positive in her blood and negative in her husband's blood and semen. Oocyte aspiration was performed, retrieving 7 oocytes, follicular fluid, and cumulus cells. RT-PCR results for the follicular fluid and cumulus cells were negative for ZIKV, and positive for only 2 oocytes. This is the first report in the literature analysing ZIKV in the follicular fluid, cumulus cells, and oocytes, and will contribute to the understanding of ZIKV infection and transmission.

## INTRODUCTION

Zika virus (ZIKV) is a member of the Flaviviridae virus family and is primarily transmitted by infected domestic mosquitoes such as *Aedes aegypti* and *Aedes albopictus* to a lesser extent. It was first isolated in 1947 from a *Rhesus* monkey in Zika forest in Uganda and was recognised in humans in 1952 in Nigeria (Dick, 1952). ZIKV infection was subsequently observed in numerous countries in Africa and Southeast Asia (Hayes, 2009; Faye *et al*., 2014). In 2007 there was the major outbreak on Yap Island in the Federated States of Micronesia, followed by French Polynesia in 2013 and 2014 (Cao-Lormeau *et al*., 2014; Lessler *et al*., 2016). In 2015 and 2016 there was an epidemic in the Americas, starting in 2015 in Brazil, and spreading throughout South America, Central America, North America and the Caribbean (Henessey *et al*., 2016).

During this outbreak of ZIKV in Brazil, an increased number of cases of newborns with microcephaly were reported in northeastern Brazil (Ministério da Saúde, 2016). In 2016, the causal association between ZIKV during pregnancy and foetal microcephaly and others cerebral anomalies was acknowledged by the World Health Organization (WHO) and the U.S. Center for Disease Control and Prevention (CDC) (WHO, 2016; Center for Disease Control and Prevention, 2016; Rasmussen *et al*., 2016).

Intrauterine and perinatal transmission of ZIKV has also been found (Besnard *et al*., 2014), and there are evidences of sexual transmission (Hills *et al*., 2016; McCarthy, 2016). These facts must be considered in reproductive-assisted treatments. Here we present a case report of a patient who contracted ZIKV during *in vitro* fertilisation (IVF). The objective of this observational study is to discover if ZIKV is present in the follicular fluid, cumulus cells and oocytes during an IVF cycle.

## CASE DESCRIPTION

### Patient

A 37-year-old female, indicated for IVF due to bilateral tubal factors, initiated the ovulation induction on day 2 of her menstrual cycle. One day later, she travelled to São Paulo on business with her mother and sister, taking the medications. She returned and underwent an ultrasound on day 5 of the treatment, showing a skin rash on her trunk, spreading to limbs. She reported that her mother and sister had the same symptoms. The patient's temperature was 37ºC, and she had mild low back pain and insignificant joint pain. A complete blood count (CBC) did not show changes in the platelets and leukocytes. The patient and her husband had a blood test for ZIKV using a reverse transcriptase polymerase chain reaction (RT-PCR). The patient offered to follow the treatment until oocyte aspiration at the clinic's expense. Oocyte aspiration was performed and seven MII stage oocytes were retrieved. The patient's follicular fluid and cumulus cells were donated for study in addition to her husband's semen.

This is a case report of a single-patient at our private IVF center, thus no Institutional Review Board (IRB) approval was obtained, because our IRB designates a single-patient case report as not subject to IRB review because it does not meet the definition of human subjects research. Nevertheless, informed written consent was taken from the couple.

## METHODOLOGY

### Ovarian stimulation protocol

A basal ultrasound was performed on the second day of the patient's cycle. Recombinant follicle-stimulating hormone (r-FSH; Gonal F, Merck Serono, Rio de Janeiro, Brazil) at a dosage of 150 IU was administered from days 2 to 4 of her menstrual cycle. Human menopausal gonadotropin (hMG; Menopur, Ferring, São Paulo, Brazil) at a dosage of 150 IU was administered from days 2 to 6. Ultrasound was performed on day 7 of the cycle and daily afterward until the administration of recombinant human chorionic gonadotropin (r-hCG; Ovidrel, Merck Serono, Rio de Janeiro, Brazil).

Gonadotrophin-releasing hormone (GnRH) antagonist (Cetrotide, Merck, Rio de Janeiro, Brazil) was initiated on day 7, when the follicles reached 16mm in diameter. When two or more follicles reached 20 mm diameter, 250 mcg of recombinant human chorionic gonadotropin (r-hCG; Ovidrel, Merck Serono, Rio de Janeiro, Brazil) was administered and oocyte aspiration was performed 36 hours later using a transvaginal ultrasound-guided needle. 

The patient had eight follicles and we collected seven MII oocytes. The oocytes, follicular fluid and cumulus corona cells were cryopreserved.

### Genetic analysis 

The blood samples obtained from the patient and her husband, the follicular fluid, cumulus cells and oocytes from the patient, and semen from her husband was tested for ZIKV RNA using a real-time reverse transcriptase polymerase chain reaction (RT-PCR). The genetic analysis was performed at the Chromosome Genomic Medicine laboratory in Sao Paulo, Brazil.

### Purification of viral RNA 

RNA was extracted from 140 µl of the patient and her husband's samples using a QIAamp Viral RNA Mini Kit (Qiagen, Hilden, Germany), following the manufacturers' protocol.

Briefly, 140 µl of samples were mixed with AVLCarrier RNA buffer. After 10 min of incubation at room temperature, 560 µl of ethanol (96-100%) was added. The samples were transferred into a column containing silica and centrifuged at 6000 x *g* for 1 min. The RNA was washed twice with buffer AW1 and AW2, respectively. RNA was eluted in 60 µl of AVE buffer and stored at -20ºC until use.

### Real-time RT-PCR

All assays were performed using the RT-PCR Kit BR ZIKV (LGC Biotechnology, Brazil) with amplification in the StepOnePlus(tm) Real-Time PCR System (Thermo-Fisher Scientific, Waltham, MA, USA), following the manufacturer's protocol.

For the real time RT-PCR, was used with a 25 µl reaction mixture under the following conditions: 12.5 µl of 2 × RT-PCR buffer, 0.5 µl of One-Step RT-PCR enzyme; 0.5 µl of primers and a BR-ZIKV probe; 0.5 µl of set of primers and a probe of the internal control (IC); 10 µl purified viral RNA (sample), and 1.5 µl nuclease-free water. The negative control consisted of blank reagent and water. Nucleic acid extracted from virus stocks was used for the positive control.

The following thermal profile was used for a single cycle of 30 min at 55ºC for reverse transcriptase, a single cycle of 30 sec at 95ºC for initial denaturation, followed by 40 amplification cycles of 10 sec at 95ºC for denaturation, 15 sec at 55ºC for annealing, and 45 sec at 68ºC for extension. The data were analysed using StepOne Plus software (SDS software from Thermo-Fisher).

## RESULTS 

The real-time RT-PCR results for ZIKV in the blood was positive for the patient, and negative for her husband, and his semen was also negative. Both the follicular fluid and cumulus cells were negative for ZIKV RNA. ZIKV RNA was present in two of the seven oocytes.

## DISCUSSION

This is the first report in the literature analysing the presence or absence of ZIKV in the follicular fluid, cumulus cells and oocytes. It will improve the understanding of the transmission and infection of this virus. ZIKV infection has been associated with foetal abnormalities such as congenital microcephaly and can lead to pregnancy loss (Miner *et al*., 2016; Plourde & Bloch, 2016). ZIKV can be present in the amniotic fluid, suggesting that the virus can cross the placental barrier (Calvet *et al*., 2016). ZIKV has also been isolated from other body fluids, as blood, urine, saliva, breastmilk, and semen (Plourde & Bloch, 2016). Sexual transmission has been described in several publications (D'Ortenzio *et al*., 2016; Plourde & Bloch, 2016). In this report, the absence of the ZIKV in the husband's semen indicated that he was not infected sexually. Studies have found that male-to-female transmission is the most common (D'Ortenzio *et al*., 2016), but there have also been reports of sexual transmission from females to males (Davidson *et al*., 2016) and from males to males (Deckard *et al*., 2016).

The presence of ZIKV RNA in the two oocytes shows the importance of testing couples seeking assisted reproduction techniques because the risk of contaminating the embryo is real.

However, only two oocytes, out of seven were infected by the virus. That could be explained, in part, by the fact that RNA quickly degrades, and we may have missed detecting the virus in the other oocytes. Furthermore, we do not know if the virus was in the zona pellucida and in the oolemma, because the PCR did not separate these two areas of the oocyte.

In conclusion, mechanisms of prevention and tests to detect the virus are of extreme importance to avoid Zika, especially during epidemics in at-risk countries.
